# Motor progression trajectories and risk of mild cognitive impairment in Parkinson's disease: A latent class trajectory model from PPMI cohort

**DOI:** 10.1111/cns.14918

**Published:** 2024-08-12

**Authors:** Xi Chen, Chentao He, Jianrui Ma, Rui Yang, Qi Qi, Ziqi Gao, Tingyue Du, Piao Zhang, Yan Li, Mengfei Cai, Yuhu Zhang

**Affiliations:** ^1^ Shantou University Medical College Shantou Guangdong Province China; ^2^ Department of Neurology, Guangdong Neuroscience Institute, Guangdong Provincial People's Hospital (Guangdong Academy of Medical Sciences) Southern Medical University Guangzhou Guangdong Province China; ^3^ School of Medicine South China University of Technology Guangzhou China; ^4^ Guangdong Cardiovascular Institute Guangdong Provincial People's Hospital, Guangdong Academy of Medical Sciences Guangzhou China; ^5^ Guangzhou Key Laboratory of Diagnosis and Treatment for Neurodegenerative Diseases Guangdong Provincial People's Hospital (Guangdong Academy of Medical Sciences) Guangzhou China

**Keywords:** latent class trajectory model, mild cognitive impairment, motor progression, Parkinson's disease

## Abstract

**Aims:**

Rare studies have investigated the association between heterogeneity of motor progression and risk of early cognitive impairment in Parkinson's disease (PD). In this study, we aim to identify distinct trajectories of motor progression longitudinally and investigate their impact on predicting mild cognitive impairment (MCI).

**Methods:**

A 5‐year cohort including 415 PD patients at baseline was collected from the Parkinson's Progression Markers Initiative. The severity of motor symptoms was evaluated using the Movement Disorder Society Unified Parkinson's Disease Rating Scale part III. The latent class trajectory model and nonlinear mixed‐effects model were used to analyze and delineate the longitudinal changes in motor symptoms. Propensity score matching (PSM) was used to minimize the impact of potential confounders. Cox proportional hazard models were applied to calculate hazard ratios for MCI, and a Kaplan–Meier curve was generated using the occurrence of MCI during the follow‐up as the time‐to‐event.

**Results:**

Two latent trajectories were identified: a mild and remitting motor symptoms class (Class 1, 33.01%) and a severe and progressive motor symptom class (Class 2, 66.99%). Patients in Class 2 initially exhibited severe motor symptoms that worsened progressively despite receiving anti‐PD medications. In comparison, patients in Class 1 exhibited milder symptoms that improved following drug therapy and a slower progression. During a 5‐year follow‐up, patients in Class 2 showed a higher risk of developing MCI compared to those in Class 1 before PSM (Log‐Rank 28.58, *p* < 0.001) and after PSM (Log‐Rank 8.20, *p* = 0.004).

**Conclusions:**

PD patients with severe and progressive motor symptoms are more likely to develop MCI than those with mild and stable motor symptoms.

## Graphical abstract

The heterogeneity of motor progression in Parkinson's disease (PD) may serve as a predictive factor for early cognitive decline. This study indicates that PD patients with severe and progressive motor symptoms are at a higher risk of developing mild cognitive impairment than those with mild and stable motor symptoms.

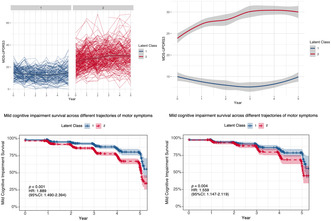

## 1 INTRODUCTION

Parkinson's disease (PD) is an insidious, progressive neurodegenerative disorder characterized by heterogeneous clinical manifestations and progression.^1^ Bradykinesia, resting tremor, and rigidity constitute the core motor syndrome of PD,^2^ with various non‐motor symptoms occurring during the disease course. Cognitive impairment, one of the most common non‐motor symptoms of PD, can develop at any disease stage and is generally accompanied by the progression of motor symptoms and the occurrence of other non‐motor symptoms.^3^ The trajectory of motor symptoms in PD varies across individuals over time, with some individuals experiencing a relatively mild course while others progressing rapidly and developing disability.^4^ More severe motor symptoms usually implicate a worse prognosis in cognitive function, resulting from a shared pathophysiology involving widespread neurotransmitter dysregulation.^3^, ^5^

Recognizing the heterogeneity of motor progression has driven the exploration of latent disease subtypes via machine learning approaches. Currently, PD subtypes have been proposed based on either single feature or multidomain phenotypes.^6^ Previous studies have concentrated on identifying subtypes primarily using cross‐sectional data, with the assumption that assigning subtypes at baseline can provide insights into disease progression.^7^, ^8^, ^9^, ^10^ However, it is necessary to consider the inherent fluctuations of the disease and the potential influence of medications administered post‐diagnosis. With the advancement in modeling techniques, the latent class trajectory model (LCTM) has been employed to analyze longitudinal data and identify subtypes with distinct progression patterns.^11^ This approach effectively overcomes the limitations of a single assessment and facilitates a more precise characterization of a patient's clinical state throughout the course of the disease.

Mild cognitive impairment (MCI), regarded as a predementia stage, is the earliest cognitive impairment that can be objectively monitored through clinical measures. Early detection and appropriate management of MCI are crucial for implementing interventions and strategies that may help slow down cognitive decline and improve overall quality of life.^12^ By delineating the trajectories of motor progression, valuable insights can be gained into the prediction of early cognitive impairment; therefore, this study aims to identify latent classes of motor symptom trajectories and investigate their impact on predicting mild cognitive impairment. The work was based on a 5‐year follow‐up dataset from the Parkinson's Progression Markers Initiative (PPMI).

## 2 MATERIALS AND METHODS

### 2.1 Study design and participants

PPMI is a multicenter, longitudinal, and observational study involving a large and highly characterized cohort, aiming to identify reliable biomarkers of PD progression. The dataset used in the present study was obtained from PPMI following formal authorization. The methodology of PPMI has been extensively described elsewhere,^13^ and all datasets were accessible at http://www.ppmi‐info.org. PPMI recruited participants who had been diagnosed with early PD within 2 years of enrollment and had not received any treatment within at least 6 months from the baseline visit. The diagnosis of early PD was eligible with only asymmetric bradykinesia or tremor plus a striatal dopamine transporter deficit on a single‐photon‐emission CT scan.^13^ This study included a 5‐year follow‐up of PD patients covering clinical information, genetic features, cerebrospinal fluid (CSF) and serum biomarkers, and cognition status. The anti‐PD drugs used during the follow‐up period were recorded in medical history, and the dosage was converted into Levodopa Equivalence Daily Dose (LEDD).

### 2.2 Clinical assessments

The severity of motor symptoms was assessed by the Movement Disorder Society Unified Parkinson's Disease Rating Scale (MDS‐UPDRS) part III. Cognitive function and other validated non‐motor symptoms related to cognition were evaluated using specific scales, including the Montreal Cognitive Assessment (MoCA) for global cognitions, the Geriatric Depression Scale Score (GDS) for depression, the State–Trait Anxiety Inventory (STAI) for anxiety, and RBD Screening Questionnaire (RBDSQ) for sleep disorder. MCI in the PPMI was determined based on Level 1 criteria that were developed by the Movement Disorder Society, including cognitive complaint by either the patient or informant (spouse, family member, or friend), at least two test scores from at least one domain with >1.0 standard deviation below the standardized mean, and no functional impairment as a result of cognitive impairment.

### 2.3 Genetic data

For the analysis of GBA and LRRK2 genes, VCFtools were used to extract variants of interest from whole exome sequencing and genome sequencing data. PLINK v1.9 was used to extract variants of interest from microarray data. Variant calls were compared and reviewed using Integrative Genomics Viewer (IGV) for discrepancies between assays. Manual curation was performed to establish a consensus variant call for each subject. The analysis of the APOE genotype was conducted using whole genome sequencing data and previously uploaded data from LONI. The results were compared with in‐house genotype data to determine the concordance and generate the final APOE allele calls for the subjects.

### 2.4 CSF and serum biomarkers detection

For the detection of CSF biomarkers, commercially available ELISA kits (Covance, Dedham, MA) were utilized to measure the level of α‐synuclein (α‐syn) in the CSF. Elecsys electrochemiluminescence immunoassays (Roche Diagnostics) were employed to detect other pathogenic proteins in the CSF, including amyloid‐beta 1–42 (Aβ42), total tau (t‐tau), and tau phosphorylated at the threonine 181 position (*p*‐tau). CSF neurofilament light chain (NfL) was quantified using Roche NTK on a Cobas e411 analyzer at Covance Greenfield laboratories. Serum NfL was measured on the Simoa singleplex NF‐light assays.

### 2.5 Statistical analysis

All statistical analysis was conducted using R (version 4.3.1). Kolmogorov–Smirnov tests were used to test the distribution of continuous variables for normality. Continuous variables were expressed as either mean (standard deviation, SD) or median [interquartile range, IQR], according to whether they followed a normal distribution. Categorical variables were expressed as frequencies and percentages. For comparison between groups, Student's *t*‐test and Mann–Whitney *U* test were done for continuous variables, and *χ* ^2^ test was done for categorical variables. Differences between groups were thought to be statistically significant when *p* < 0.05.

To identify latent classes of motor symptom trajectories in PD patients, we used the LCTM to analyze the repeatedly measured data (R package of LCTMtools). LCTM, which has been described elsewhere in detail,^11^ is a type of finite mixture modeling that aims to explore latent classes of individuals who exhibit similar progression patterns of a determinant over time or with age. Based on previous literature^11^, ^14^ and the clinical rationale for grouping, *K* = 5 was chosen as the initial classification for constructing the model pool. The best model was chosen based on a minimum Bayesian Information Criterion (BIC) while ensuring that the posterior probabilities for each class remained above 0.70 and that the class size was at least 2% of the population. In the present model, the motor evaluation scale of MDS‐UPDRS part III was chosen as the determinant, and the time from the baseline to the follow‐up period was selected as the time indicator. To account for the potential effect of anti‐PD drugs on motor progression, LEDD at each follow‐up visit was included in the mixture model. A nonlinear mixed‐effects model was conducted to fit the changes in motor symptoms by the assigned classes, generating an estimated trend and 95% confidence intervals.

To address potential selection bias and ensure a balanced comparison between groups, propensity score matching (PSM) was employed. Propensity scores were calculated by logistic regression with covariates that were statistically significant (*p* < 0.05) at baseline. Groups were matched in a 1:1 ratio using the nearest neighbor method and a caliper of 0.2. Survival probabilities before and after PSM were estimated with the Kaplan–Meier curves and compared by the log‐rank (Mantel‐Cox) test.

To assess the risk of MCI, we computed hazard ratios for MCI by the assigned classes using the Cox proportional hazard model, with validated clinical predictors of cognitive impairment included as covariates. Significant variables (*p* < 0.05) in the univariable model were included in the multivariable Cox proportional hazard model. For the survival model, time zero was the date of enrollment (baseline visit), and time‐to‐event was the first occurrence of MCI during the follow‐up period.

## 3 RESULTS

### 3.1 Demographic and clinical characteristics of participants

A total of 415 participants with available MDS‐UPDRS part III scores at baseline were included in the statistical analysis. Throughout the 5‐year follow‐up period, the number of participants who completed the follow‐up each year was as follows: 389, 373, 361, 342, and 311, respectively. Overall, the study participants had a mean age of 61.61 years (SD: 9.77), with 65.1% of them being male. All participants were newly diagnosed with PD and drug‐naive, with a median disease duration of 4.23 months (IQR: 2.52–7.80) and a median year of education of 16 years (IQR: 14–18). Table 1 provided additional detailed information on the participants' characteristics at baseline.

**TABLE 1:** Demographic and clinical characteristics of patients at baseline.

	Patients (*n* = 415)
Male	270 (65.1%)
Age, years	61.61 (9.77)
Disease onset, years	59.63 (10.04)
Duration, years	4.23 [2.52, 7.80]
Education, years	16.00 [14.00, 18.00]
MDS‐UPDRS1	5.00 [3.00, 7.00]
MDS‐UPDRS2	5.00 [3.00, 8.00]
MDS‐UPDRS3	20.00 [14.50, 26.00]
MoCA	28.00 [26.00, 29.00]
GDS	2.00 [1.00, 3.00]
STAI	61.50 [51.00, 75.00]
RBDSQ	3.00 [2.00, 6.00]
APOE ε4 carrier	104 (25.1%)
GBA carrier	54 (13.0%)
LRRK2 carrier	122 (29.4%)
CSF Aβ42	844.40 [616.32, 1123.00]
CSF α‐syn	1390.50 [1059.40, 1785.47]
CSF t‐tau	157.75 [126.97, 202.33]
CSF *p*‐tau	13.48 [11.23, 17.26]
CSF NfL	86.52 [65.97, 118.15]
Serum NfL	11.50 [8.28, 15.65]

Abbreviations: Aβ42, amyloid β 1–42; CSF, cerebrospinal fluid; GDS, Geriatric Depression Scale Score; MDS‐UPDRS, Unified Parkinson's Disease Rating Scale; MoCA, Montreal cognitive assessment; NfL, neurofilament light chain; RBDSQ, REM Sleep Behavior Disorder Questionnaire Score; STAI, State–Trait Anxiety Inventory.

### 3.2 Latent class analysis of longitudinal changes in motor symptoms

**FIGURE 1:**
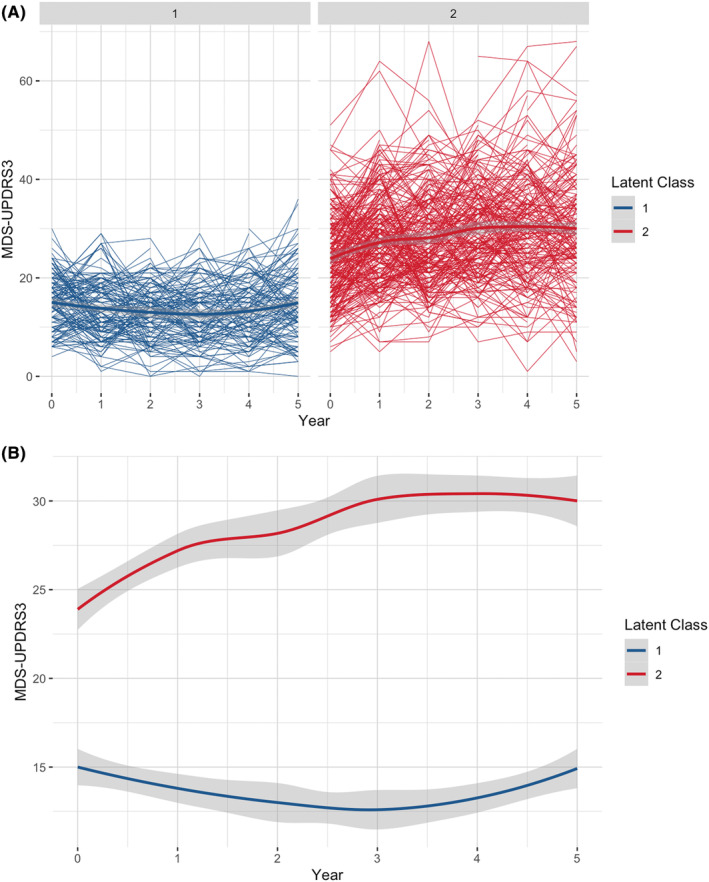
Two latent classes of longitudinal changes in motor symptoms. (A) The raw data for individual trajectories in each identified latent class. (B) Raw data were fitted and represented by solid lines, with gray‐shaded areas indicating the 95% confidence interval.

Based on the longitudinal changes in MDS‐UPDRS part III scores, participants were categorized into classes using the follow‐up period as the time indicator. The results of LCTM construction are presented in Tables 2, 3, 4. Table 2 presented the results of BIC testing and Tables 3 and 4 presented the results of model adequacy assessments, including posterior probabilities and classification proportion. For the five evaluated models, only the two‐class model exhibited the best fit with the lowest BIC meanwhile the posterior probabilities were above 0.70 and the sample size in each class was above 2%. Thus, the two‐class model was chosen as an optimal model. Figure 1 illustrates individual trajectories within the two latent classes (Figure 1A) and the corresponding fitted trends with 95% confidence intervals (Figure 1B). In this model, Class 1 included 137 participants (33.01%) who exhibited low initial MDS‐UPDRS part III scores and then remitted, which could be due to the intervention of anti‐PD drugs. The overall scores remained low and relatively stable throughout the follow‐up period (i.e., remitting motor symptom class). In contrast, Class 2 included 278 participants (66.99%) with high initial MDS‐UPDRS part III scores that progressively increased over time (i.e., progressive motor symptom class).

**TABLE 2:** Model fit evaluation information for each LCTM tested.

	BIC
One‐class LCTM	14936.45
Two‐class LCTM	14902.54
Three‐class LCTM	14911.79
Four‐class LCTM	14937.40
Five‐class LCTM	14961.80

Abbreviations: BIC, Bayesian information criteria; LCTM, latent class trajectory model.

**TABLE 3:** Posterior probabilities in each LCTM tested.

	Class 1	Class 2	Class 3	Class 4	Class 5
Two‐class LCMM	0.8091	0.9067	–	–	–
Three‐class LCMM	0.8165	0.8345	0.8313	–	–
Four‐class LCMM	0.8182	0.6102	0.8121	0.8387	–
Five‐class LCMM	0.8258	0.7763	0.6299	0.8373	0.8183

Abbreviation: LCTM, latent class trajectory model.

**TABLE 4:** Posterior classification proportion in each LCTM tested.

	Class 1	Class 2	Class 3	Class 4	Class 5
Two‐class LCMM	33.01%	66.99%	–	–	–
Three‐class LCMM	39.28%	55.90%	4.82%	–	–
Four‐class LCMM	41.20%	5.78%	49.88%	3.13%	–
Five‐class LCMM	36.87%	2.17%	5.54%	53.49%	1.93%

Abbreviation: LCTM, latent class trajectory model.

### 3.3 Comparison between two latent classes before and after PSM

The baseline characteristics of participants between two latent classes before and after PSM were compared in Table 5. Before PSM, compared with Class 1, participants in Class 2 exhibited an older average age [59.17 (9.99) vs. 62.81 (9.45), *p* < 0.001] and age of disease onset [57.39 (10.29) vs. 60.74 (9.74), *p* = 0.001], with higher education levels (16.00 [13.00, 17.00] vs. 16.00 [14.00, 18.00], *p* = 0.026) but lower global cognition (28.00 [26.00, 29.00] vs. 27.00 [26.00, 29.00], *p* = 0.016). The overall performance on MDS‐UPDRS in Class 2 was worse than in Class 1 (*p* < 0.05). As for biomarkers, participants in Class 2 had a lower level of CSF Aβ42 (883.20 [655.10, 1237.00] vs. 828.40 [594.10, 1051.00], *p* = 0.017) but a higher level of Serum NfL (10.40 [7.86, 14.10] vs. 12.00 [8.62, 16.45], *p* = 0.005) compared to those in Class 1. There were no statistical significances between the two classes in gender, disease duration, emotion, sleep, or genetic features at baseline. PSM was conducted with propensity scores calculating for the above significant covariates, yielding a total of 102 pairs. All baseline characteristics were not statistically significant between the two classes (*p* > 0.05). Comparisons of LEDD between classes for 5 years were shown in Table 6 and no statistical significances were found (*p* > 0.05).

**TABLE 5:** Baseline demographic differences between two latent classes before and after matching.

	Before PSM		*p*	After PSM		*p*
	Class 1 (*N* = 137)	Class 2 (*N* = 278)		Class 1 (*N* = 102)	Class 2 (*N* = 102)	
Male	82 (59.9%)	188 (67.6%)	0.146	60 (58.8%)	65 (63.7%)	0.565
Age, years	59.17 (9.99)	62.81 (9.45)	**<0.001**	60.58 (9.63)	60.68 (9.80)	0.942
Disease onset, years	57.39 (10.29)	60.74 (9.74)	**0.001**	58.65 (10.06)	58.71 (10.31)	0.969
Duration, years	3.70 [2.47, 6.57]	4.38 [2.53, 9.13]	0.224	4.45 [2.68, 6.95]	4.05 [2.47, 8.22]	0.693
Education, years	16.00 [13.00, 17.00]	16.00 [14.00, 18.00]	**0.026**	16.00 [13.00, 18.00]	16.00 [13.25, 17.00]	0.980
MDS‐UPDRS1	4.00 [2.00, 6.00]	5.00 [3.00, 8.00]	**0.001**	5.00 [2.00, 6.00]	4.00 [2.00, 7.75]	0.799
MDS‐UPDRS2	4.00 [2.00, 6.00]	5.00 [3.00, 9.00]	**<0.001**	4.00 [2.00, 7.00]	4.00 [2.00, 6.00]	0.589
MDS‐UPDRS3	15.00 [11.00, 19.00]	23.00 [17.00, 29.75]	**<0.001**	16.50 [14.00, 20.00]	17.00 [13.00, 22.00]	0.505
MoCA	28.00 [26.00, 29.00]	27.00 [26.00, 29.00]	**0.016**	28.00 [26.00, 29.00]	28.00 [26.00, 29.00]	0.537
GDS	2.00 [0.00, 3.00]	2.00 [1.00, 3.00]	0.117	1.50 [0.00, 3.00]	2.00 [1.00, 3.00]	0.418
STAI	62.00 [51.00, 73.00]	61.00 [51.00, 75.00]	0.431	62.00 [51.00, 72.50]	60.00 [49.00, 73.50]	0.680
RBDSQ	3.00 [2.00, 5.00]	4.00 [2.00, 6.00]	0.177	3.00 [2.00, 5.00]	4.00 [2.00, 5.75]	0.101
APOE ε4 carrier	28 (20.4%)	76 (27.3%)	0.160	18 (17.6%)	26 (25.5%)	0.233
GBA carrier	19 (13.9%)	35 (12.6%)	0.834	14 (13.7%)	12 (10.8%)	0.669
LRRK2 carrier	39 (28.5%)	83 (29.9%)	0.859	31 (30.4%)	37 (36.3%)	0.458
CSF Aβ42	883.20 [655.10, 1237.00]	828.40 [594.10, 1051.00]	**0.017**	874.60 [654.43, 1185.00]	848.60 [614.52, 1075.00]	0.569
CSF α‐syn	1412.20 [1085.50, 1865.60]	1374.50 [1051.40, 1761.60]	0.367	1428.15 [1087.20, 1836.85]	1455.60 [1129.53, 1814.35]	0.863
CSF t‐tau	156.80 [128.62, 207.20]	158.10 [126.55, 195.48]	0.935	155.70 [129.12, 201.57]	161.75 [134.28, 192.95]	0.531
CSF *p*‐tau	13.29 [11.18, 17.67]	13.53 [11.25, 17.16]	0.875	13.61 [11.22, 17.10]	13.98 [11.51, 15.81]	0.802
CSF NfL	83.62 [66.88, 112.80]	89.34 [65.91, 119.60]	0.518	101.76 [87.75, 101.76]	101.76 [78.20, 101.76]	0.593
Serum NfL	10.40 [7.86, 14.10]	12.00 [8.62, 16.45]	**0.005**	11.05 [8.96, 14.45]	11.55 [8.22, 13.88]	0.602

*Note*: Bold indicates statistical significance.

Abbreviations: Aβ42, amyloid β 1–42; CSF, cerebrospinal fluid; GDS, Geriatric Depression Scale Score; MDS‐UPDRS, Unified Parkinson's Disease Rating Scale; MoCA, Montreal cognitive assessment; NfL, neurofilament light chain; RBDSQ, REM Sleep Behavior Disorder Questionnaire Score; STAI, State–Trait Anxiety Inventory.

**TABLE 6:** LEDD comparison between two latent classes before matching.

	Follow‐up	Class 1	Class 2	*p*
LEDD, mg	Baseline	0	0	–
	Year 1	152.00 [0.00, 300.00]	100.00 [0.00, 300.00]	0.098
	Year 2	300.00 [150.00, 500.00]	300.00 [100.00, 440.00]	0.156
	Year 3	400.00 [195.00, 600.00]	400.00 [205.00, 599.00]	0.639
	Year 4	500.00 [300.00, 700.00]	450.00 [300.00, 650.00]	0.604
	Year 5	400.00 [195.00, 600.00]	400.00 [205.00, 599.00]	0.639

Abbreviation: LEDD Levodopa equivalence daily dose.

### 3.4 Associations between motor progression and mild cognitive impairment

**FIGURE 2:**
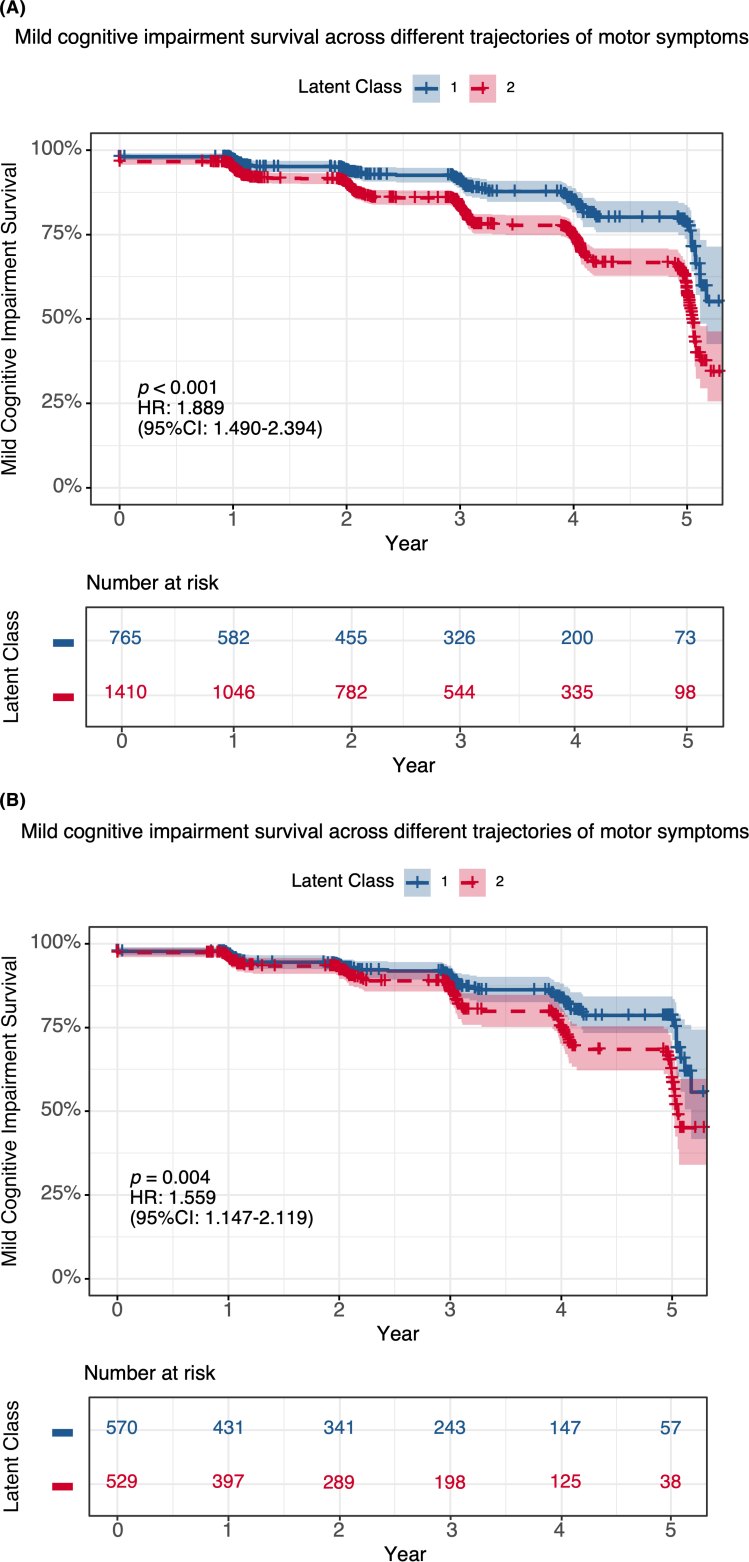
The risk of developing mild cognitive impairment between two distinct trajectories of motor symptoms before (A) and after (B) propensity score matching. (A) The Kaplan–Meier survival curve showed that patients in Class 2 had a higher risk of developing mild cognitive impairment than those in Class 1 before propensity score matching (*p* < 0.001). (B) Similarly, the survival rates remained comparable with significant differences detected after propensity score matching (*p* = 0.004).

The relationship between latent classes of motor progression trajectories and mild cognitive impairment was assessed through Kaplan–Meier curves and log‐rank (Mantel‐Cox) test. Significant differences were revealed both before (Log‐Rank 28.58, *p* < 0.001, Figure 2A) and after (Log‐Rank 8.20, *p* = 0.004, Figure 2B) PSM. The 5‐year MCI survival rate between classes was 78.6% to 57.4% [HR (95% CI): 1.889 (1.490–2.394)] before PSM and 78.6%–58.4% [HR (95% CI): 1.559 (1.147–2.119)] after PSM. These findings suggested that participants with progressive motor symptoms over a 5‐year follow‐up period had a significantly higher risk of developing MCI compared to those with remitting and stable motor symptoms.

Potential predictors of cognitive impairment were included as covariates in the Cox proportional hazard model to help identify the class with a higher risk for MCI. The results of the univariable and multivariable Cox proportional hazard model are presented in Table 7. In the univariable model, the conversion from normal cognition to MCI was not found to be associated with factors such as disease duration, MDS‐UPDRS part II scores, CSF α‐synuclein, CSF total tau, CSF phosphorylated tau, and CSF NfL levels. However, anxiety (assessed by STAI, HR = 1.016, 95% CI 1.003–1.029, *p* = 0.019) emerged as an independent predictor of mild cognitive impairment in both the univariate and multivariate models. The multivariable model included additional established predictors of MCI, such as years of education, MoCA scores, MDS‐UPDRS part I, LRRK2 gene status, CSF Aβ42, and serum NfL levels.

**TABLE 7:** Cox proportional hazard analysis for the predictors of mild cognitive impairment.

Variables	Univariable analysis		Multivariable analysis	
	HR (95% CI)	*p*	HR (95% CI)	*p*
Male	1.587 (1.256–2.004)	**<0.001**	0.883 (0.592–1.316)	0.541
Age	1.018 (1.007–1.029)	**<0.001**	0.900 (0.778–1.042)	0.159
Disease onset	1.020 (1.009–1.031)	**<0.001**	1.128 (0.976–1.304)	0.102
Duration	1.002 (0.987–1.018)	0.781		
Education	0.931 (0.899–0.963)	**<0.001**	0.923 (0.860–0.992)	**0.030**
MDS‐UPDRS1	1.028 (1.011–1.046)	**0.002**	0.936 (0.887–0.988)	**0.016**
MDS‐UPDRS2	1.011 (0.995–1.028)	0.187	1.003 (0.953–1.057)	0.889
MDS‐UPDRS3	1.017 (1.008–1.025)	**<0.001**	1.015 (0.998–1.032)	0.087
MoCA	0.821 (0.804–0.839)	**<0.001**	0.836 (0.797–0.879)	**<0.001**
GDS	1.105 (1.072–1.138)	**<0.001**	1.053 (0.956–1.159)	0.298
STAI	1.019 (1.014–1.024)	**<0.001**	1.016 (1.003–1.029)	**0.019**
RBDSQ	1.070 (1.037–1.104)	**<0.001**	1.043 (0.982–1.108)	0.170
APOE ε4 carrier	1.285 (1.025–1.610)	**0.029**	0.818 (0.537–1.248)	0.352
GBA carrier	1.415 (1.080–1.851)	**0.012**	1.200 (0.731–1.968)	0.471
LRRK2 carrier	0.523 (0.404–0.677)	**<0.001**	0.596 (0.388–0.915)	**0.018**
CSF Aβ42	0.999 (0.999–1.000)	**<0.001**	0.999 (0.999–1.000)	**0.009**
CSF α‐syn	1.000 (0.9996–1.000)	0.173		
CSF t‐tau	0.999 (0.997–1.001)	0.438		
CSF *p*‐tau	0.998 (0.971–1.025)	0.860		
CSF NfL	1.001 (1.000–1.003)	0.103		
Serum NfL	1.003 (0.994–1.013)	0.495	0.965 (0.937–0.993)	**0.016**

*Note*: Bold indicates statistical significance.

Abbreviations: Aβ42, amyloid β 1–42; CSF, cerebrospinal fluid; GDS, Geriatric Depression Scale Score; MDS‐UPDRS, Unified Parkinson's Disease Rating Scale; MoCA, Montreal cognitive assessment; NfL, neurofilament light chain; RBDSQ, REM Sleep Behavior Disorder Questionnaire Score; STAI, State–Trait Anxiety Inventory.

## 4 DISCUSSION

Based on a large longitudinal cohort of PD patients from PPMI, we identified a two‐class model that charts the trajectories of motor symptoms in the consideration of medication usage. Class 1, characterized by mild initial motor symptoms that remitted and remained stable throughout the course of the disease, exhibited a lower likelihood of developing MCI. Conversely, Class 2, distinguished by more severe initial motor symptoms that worsened over time, consistently demonstrated a higher risk of developing MCI at every follow‐up. The conclusion remained consistent after balancing baseline characteristics by PSM.

Growing clinical evidence has substantiated the possible link between motor disability and cognitive dysfunction in PD patients, and related studies have indicated that these two factors serve as mutual predictors. It has been suggested that cognitive profile at baseline is associated with motor prognosis and poor cognition may predict rapidly deteriorated motor symptoms.^15^, ^16^ On the contrary, PD patients with axial motor impairments, such as postural instability or gait difficulty, generally present with more severe motor symptoms and are more relevant to a faster progression to cognitive dysfunction.^17^, ^18^ All of these studies have relied on a single assessment at the baseline to evaluate the disease status and predict disease prognosis, overlooking the potential fluctuations and confounding factors that may occur during the course of the disease. Our study, on the other hand, not only reaffirmed the findings of these previous studies but also supported them from both a modeling perspective and a longitudinal standpoint. Shared or parallel pathophysiology may be the potential mechanism for this relationship. In PD, the primary cause of motor symptoms is dopaminergic degeneration in the substantia nigra and striatum. However, when the associative dorsal caudate nucleus is affected, it may also be implicated in MCI. More extensive dopaminergic degeneration affecting the frontal, temporal, and parietal cortical regions is associated with the occurrence of dementia.^3^, ^19^ Of note, cholinergic disturbances in the basal forebrain not only independently contribute to cognitive decline but also interact with the more pronounced loss of dopamine in the caudate nucleus, leading to a more significant cognitive decline.^20^, ^21^

Higher educational attainment has consistently been considered a protective factor for cognitive decline.^22^ Previous studies have revealed that higher education could increase resilience against the detrimental effects of progressive neuropathological accumulation in Alzheimer's dementia^23^, ^24^ meanwhile exerting protective effects in the later stages of PD.^25^ At baseline, we observed that individuals with progressed trajectories, in comparison to those with stable trajectories, exhibited more severe initial motor symptoms. Interestingly, the class with progressed trajectories had higher levels of education; however, they exhibited lower overall performance on global cognition despite not meeting the criteria for cognitive impairment. Furthermore, the Cox model confirmed that both higher educational levels and higher scores on the MoCA were protective factors against the development of mild cognitive impairment. These findings imply that more severe motor symptoms may serve as an indication of poorer cognitive function, both in terms of cross‐sectional and longitudinal perspectives. Mutations in the LRRK2 gene are the most common cause of monogenic PD.^26^ Previous studies have consistently reported that LRRK2 mutation carriers with PD exhibit slower cognitive decline or milder cognitive symptoms than non‐carriers.^27^, ^28^, ^29^ In line with these findings, our current study sheds further light on the protective role of LRRK2 mutation carriers in cognition.

Aβ42 is derived from the amyloid precursor protein, and its pathological aggregation results in the formation of amyloid plaques, whose deposition can cause neuronal damage, ultimately leading to cognitive impairment.^30^ This neuropathological process can be reflected by a decline in Aβ42 level in the CSF. Numerous studies suggested that a reduction in CSF Aβ42 level serves as a promising biomarker for predicting cognitive decline in PD.^31^, ^32^ Consistent with these studies, we observed a protective effect of CSF Aβ42 level in the development of cognitive impairment among patients with more severe motor symptoms. In addition, emerging evidence supports that Aβ pathology may play a role in locomotor function.^33^ Our study drew the same conclusion that low CSF Aβ42 levels at the baseline are associated with progressed trajectories of motor symptoms. Furthermore, several studies have demonstrated that increased plasma NfL levels are associated with a more severe phenotype of PD, faster motor progression, and accelerated cognitive decline.^34^, ^35^ Aligning with previous research, our study confirmed that serum NfL level is a risk factor for the development of cognitive impairment. Although statistically significant differences were found in serum NFL levels between distinct motor progression classes at baseline, it cannot be determined whether this difference was related to motor progression. Further investigation on this aspect is needed.

Anxiety is highly prevalent in PD patients and has been widely recognized as an independent risk factor for cognitive decline.^36^, ^37^ In our study, we observed the prognostic effect of anxiety in identifying individuals with a higher risk of developing mild cognitive impairment. These findings have been demonstrated by different methodologies and study cohorts. Previous research has consistently validated a significant association between anxiety and cognitive functioning, with anxiety being positively correlated with the development of cognitive impairment.^38^, ^39^ Moreover, it has been revealed that anxiety might be linked explicitly to even earlier stages of cognitive decline, such as subjective cognitive complaints.^40^ The underlying mechanisms for this association remain unclear. One possible explanation might be PD‐related alteration of the fear circuit and the limbic cortical‐striatal‐thalamocortical circuits. Anatomically, there is an essential overlap between these two circuits. On the one hand, in PD patients, the basal ganglia dysfunction and hypo‐dopaminergic state can cause the fear circuit to be overactive, leading to disruption in fear processing. Meanwhile, the limbic cortical‐striatal‐thalamocortical circuit may be underactive, affecting long‐term cognitive and behavioral adaptation to fear.^41^ On the other hand, altered levels of neurotransmitters, including dopamine, serotonin, and catecholamines, in the thalamus and locus coeruleus, were observed in the early stages of PD using positron emission tomography or single‐photon emission computed tomography.^41^, ^42^, ^43^ These changes can potentially contribute to dysfunction in both circuits, either occurring simultaneously or sequentially. This also explains the early occurrence and high prevalence of anxiety in PD patients and an associated elevated risk of cognitive impairment subsequently.

The limitations of this study need to be acknowledged. First, flaws in the latent class trajectory model result in the underestimation of variance. Potential challenges associated with relatively small sample sizes include difficulties in obtaining reliable fit indices, uncovering classes with low membership and convergence issues. Increasing the sample size can effectively address these issues. Second, as this was a longitudinal cohort study with a 5‐year follow‐up period, some rating scales and questionnaires were missing due to the extended duration of follow‐up. Additionally, PD is a progressive neurodegenerative disease, and relying solely on 5 years of data may not provide a comprehensive understanding of disease progression. Long‐term follow‐up is crucial for further analysis. Furthermore, although the study considered the effect of anti‐PD drugs, other potential confounding variables, such as ethnicity, antipsychotic drugs, comorbidities, disparities in care, and lifestyle, may not have been adequately controlled for.

In conclusion, this study identified two distinct trajectories of motor progression in de novo PD patients. The motor progressed class exhibited a higher risk of developing MCI. Educational attainment, global cognition, CSF Aβ42 level, serum NfL level, and anxiety helped to identify the class with increasing risk for MCI. These findings highlight the importance of considering motor progression patterns in predicting cognitive impairment in PD. Early detection of motor progression subgroups may aid in implementing interventions to slow down cognitive decline and improve patients' quality of life. Further research is needed to explore the underlying mechanisms and develop targeted interventions for different motor progression subtypes in PD.

## AUTHOR CONTRIBUTIONS

XC, CH, JM, RY, QQ, ZG, and TD conducted the study. XC, CH, and RY extracted and analyzed the data. XC wrote the first draft of the manuscript. JM, PZ, YL, MC, and YZ reviewed the manuscript and provided suggestions for revision. All authors approved the final version for publication.

## CONFLICT OF INTEREST STATEMENT

None of the authors have any financial disclosures or conflicts of interest.

## Data Availability

Data used in this article were obtained from the Parkinson's Progression Markers Initiative (PPMI) database (https://www.ppmi‐info.org/access‐data‐specimens/download‐data), RRID:SCR_006431. For up‐to‐date information, please visit www.ppmi‐info.org.
